# Cover crops and compost prevent weed seed bank buildup in herbicide‐free wheat–potato rotations under conservation tillage

**DOI:** 10.1002/ece3.4942

**Published:** 2019-02-07

**Authors:** Jan H. Schmidt, Stephan Junge, Maria R. Finckh

**Affiliations:** ^1^ Group of Ecological Plant Protection University of Kassel Witzenhausen Germany

**Keywords:** compost, conservation agriculture, cover crop, organic management, reduced tillage, seed bank, weed dynamic

## Abstract

1. Weeds are a major constraint affecting crop yields in organic farming and weed seed bank analysis can be an important tool for predicting weed infestation and assessing farming system sustainability.
2. We compared the weed seed banks two and four years after transition from conventional to reduced tillage in organically managed winter wheat–potato cropping sequences in two replicated field trials. Experimental factors were either conventional (CT) with moldboard (25 cm) or reduced tillage (RT) with chisel ploughing (5–15 cm). Dead mulch (8–10 cm), consisting of rye–pea or triticale–vetch mixtures, was additionally applied to potatoes in the RT system. In both systems, one‐half of the plots received 5 t (ha/year) dry matter of a commercially sold yard waste compost as an organic amendment. Furthermore, subsidiary crops were grown in both systems, either as legume living mulches undersown in wheat or as cover crops sown after wheat. Prior to sowing the wheat and after potatoes, the soil seed bank from 0 to 12.5 and from 12.5 to 25 cm was sampled and assessed in an unheated glasshouse over nine months.
3. The initial weed seed bank size in the topsoil was uniform (4,420 seedlings m^−2^). Two years later, wheat‐associated weeds, such as *Galium aparine, Lamium *spp., and *Myosotis arvensis, *were 61% higher on average in RT than in CT. This was independent of subsidiary crops used. In contrast, *Chenopodium album, *a potato‐associated weed that depends on intensive tillage, was reduced by 15% in the mulched RT system compared to CT. When RT was combined with cover crops and compost application, the seed bank did not differ significantly from the CT system.
4. We conclude that subsidiary crops, mulches, and potentially compost are important management tools that contribute to the success of RT in herbicide‐free cereal‐based systems in temperate climates.

## 1 INTRODUCTION

The adoption and promotion of conservation agriculture can greatly reduce agricultural pollution caused by nitrogen leaching, soil erosion, and excessive diesel consumption (Köller, 2003). While conservation agriculture is broadly applied in North and South America, its use is limited in Europe (Kassam, Friedrich, & Derpsch, 2010), especially in organic systems due to the generally higher weed pressure (Peigné, Ball, Roger‐Estrade, & David, 2007).

Especially organic rotations in Europe are still based on deep soil‐inversion tillage aiming for weed suppression, which generally undermines the sustainability of agricultural systems. In detail, deep soil‐inversion tillage increases erosion risks and organic matter decay, disturbs soil communities adapted to specific soil depths, disrupts arbuscular mycorrhizal networks, and decimates earthworm populations (Carr, Gramig, & Liebig, 2013; Gosling, Hodge, Goodlass, & Bending, 2006; Tebrügge & Düring, 1999). Organic farmers have to learn to manage their systems with a minimum of tillage if they truly aim for long‐term sustainability. Besides appropriate crop rotations, nonchemical weed suppression can be achieved by the introduction of high biomass producing cover crops in the rotation (Mirsky et al., 2012), the use of weed‐suppressive composts (Blackshaw, Molnar, & Larney, 2005; Ozores‐Hampton, Obreza, & Stoffella, 2001), or the application of surface mulches (Campiglia, Radicetti, & Mancinelli, 2015).

To determine long‐term effects of such management operations on weeds, not only the weed vegetation in the field but also weed seed banks need to be considered. Although both parameters generally correlate, weed seeds can buffer short‐ and long‐term cropping system effects due to their longevity and are therefore better indicators for long‐term system effects than the aboveground vegetation (Mayor & Dessaint, 1998).

While several studies have documented the effects of crop rotation on weed pressure in organic farming in the short or medium term (Albrecht, 2005; Menalled, Gross, & Hammond, 2001; Sjursen, 2001; Teasdale, Mangum, Radhakrishnan, & Cavigelli, 2004), information about the effects of noninversion tillage on the soil weed seed bank dynamics over time is scant. A German study showed that chisel ploughing resulted in twofold higher weed seed banks compared with deep ploughing 5 years after differential tillage was started (Gruber & Claupein, 2009). However, initial seed bank densities and seed banks of single annual weed species 5 years after differential tillage were not examined, thus preventing any conclusions about seed bank dynamics. To obtain such information, there is a need to follow the transition process to noninversion tillage from the beginning preferably in comparison with a conventionally tilled system.

Two experiments were set up in 2010 and 2011 to study the transition and longer term effects of conservation agriculture in adjacent fields, managed organically since 1989. The experiments were embedded in a cropping sequence starting with 2 years of grass‐clover followed by winter wheat and potato. A typical plough‐based system is compared with a noninversion tillage system that includes applications of transfer mulch to potatoes. The second factor was the application of living mulches in winter wheat compared with cover crops sown after wheat harvest. The third factor was yard waste compost application compared with mineral fertilization.

In this study, the development of the weed seed banks in two tillage, two cover crop, and two fertilizer systems 4 years after the start of the experiments is reported. Therefore, the viable weed seed bank was assessed after potatoes in 2014 and 2015 covering the minimally tilled horizon (0–12.5 cm) and the horizon reached by ploughing (12.5–25 cm) and compared with the initial weed seed bank in 2012 and 2013 (data unpublished). The specific aims of the study were to determine (a) quantitative and compositional changes in the weed seed bank over the course of the wheat–potato cropping sequence under mulch‐based noninversion tillage compared to ploughing; (b) the effect of specific agricultural management options (noninversion vs. plough tillage, living mulches vs. cover crops, compost vs. no compost) adapted for organic farming on the viable weed seed bank; and (c) interactions among these systems with respect to the weed seed bank.

## 2 MATERIAL AND METHODS

Experiments were set up in 2010 and in 2011 in adjacent fields located on the organic experimental farm of the University of Kassel in Neu‐Eichenberg (51°22′51″N, 9°54′44″E, 231 m ASL with an eastern incline of 3%). The soil type is a Haplic Luvisol with 3.3% sand, 83.4% silt, and 13.3% clay (USDA classification Zc). Both experiments started with two years of grass‐clover, which was mulched repeatedly, followed by winter wheat and potato. In the years preceding the grass‐clover, the soil had been regularly ploughed 20–25 cm deep. The experiments consisted of a split‐split‐plot design with four replicates. The main factor (12 × 60 m^2^) was noninversion tillage by chisel ploughing (5–15 cm) including the application of dead mulch to potatoes (RT) versus conventional tillage based on moldboard ploughing (CT, 20–25 cm) to terminate the grass‐clover and subsidiary crops. For the second factor “crop rotation,” each tillage main plot (12 × 60 m^2^) was split into two 6 × 60 m^2^ subplots. Two clover species were undersown in the winter wheat as “living mulch” (*Trifolium repens* L. and *T. subterraneum* L.) in one of the subplots while either summer vetch (*Vicia sativa* L.) or an oilseed radish/black oat mixture (*Raphanus sativus* L., *Avena strigosa* L.) was sown as cover crops after wheat harvest in the remaining subplot. The direct drilling of cover crops was accompanied by shallow undercutting (4 cm) with 36 cm overlapping duck‐foot shares. For the third factor “fertilization,” each living mulch/cover crop subplot (6 × 60 m^2^) was split into four 6 × 15 m^2^ sub‐subplots resulting in two sub‐subplots per living mulch/cover crop species per main plot. In one of these sub‐subplots per living mulch/cover crop species, 5 t and 10 t dry matter ha^−1 ^yard waste compost were applied manually after soil tillage before sowing wheat and planting potatoes, respectively. Before planting potatoes, the remaining sub‐subplot per living mulch/cover crop species received potassium (K_2_SO_4_) and phosphorus (rock phosphate) fertilizer approximately matching the concentration of the composts used in 2014 and 2015 (Table 1). In total, the experiments consisted of 64 plots (4 replicates × 2 tillage treatments × 4 living mulch/cover crop species × 2 fertilizer treatments). However, both living mulch species in wheat largely failed in both experiments due to winterkill and were treated as weedy fallow in the analysis. In addition, the two cover crops performed poorly due to late sowing in the first and unfavorable sowing conditions paired with a common vole (*Microtus arvalis *Pallas) epidemic in the second experiment and were combined into one cover crop treatment, thus resulting in eight weedy fallow–cover crop comparisons per experiment.

**Table 1:** Chemical characteristics, including the composting duration (age), dry matter (DM), bulk density (BD), pH, electrical conductivity (EC), potassium (K), phosphorous (P), total nitrogen (N_t_), carbon (C_t_), and C/N ratio of yard waste composts (≤20 mm sieved) from municipal trees and shrubbery from the composting plant at Dransfeld, Germany (three‐month‐old) used in 2012 and 2013 before wheat, and from a composting plant near Hannover (Aha, nine‐month‐old) used before potatoes in 2014 and 2015

Year/crop	Age (month)	DM (%)	BD (g/L)	pH	EC (Μs/cm)	K (mg/kg)	P (mg kg)	N_t_ (%)	C_t_ (%)	C/N ratio
2012/Wheat	3	85	389	7.5	498	3,104	541	1.8	29.0	16.0
2013/Wheat	3	81	282	6.4	778	NA	807	1.5	37.4	25.5
2014/Potato	9	75	604	7.3	915	5,276	547	1.3	20.8	16.2
2015/Potato	9	60	731	8.1	1,011	4,858	616	1.3	16.9	13.0

Potatoes were planted in late April and received an 8–10 cm layer of rye–pea (2014; 12 t/ha dry matter; C:N = 27) and triticale–vetch (2015; 26.5 t/ha dry matter; C:N = 23) transfer mulch three weeks after planting in the RT treatments after the first hilling. No further tillage was applied to the RT plots while potatoes in CT plots were harrowed and hilled once more in June. If not already killed by late blight (*Phytophthora infestans*), potatoes and weeds were mulched in early August and harvested in early September.

### 2.1 Weed seed bank analysis

Twenty 2.4‐cm‐diameter soil cores were taken from each sub‐subplot after seedbed preparation and compost application, but before sowing of winter wheat (start of the experiment in 2012 and 2013) and after potato harvest (end of the experiment in 2014 and 2015). Soil cores were divided into 0–12.5 cm and 12.5–25 cm layers. Plastic trays (200 cm^2^) were filled with 600 ml soil and placed in an unheated greenhouse with a plastic roof (coldhouse). There were 128 trays representing the trial plus four pure compost controls. The soil in the trays was kept moist, but watering was suspended when frost occurred. From October until June, emerged seedlings were periodically identified, counted, and removed from plastic trays. Vegetative reproductive parts of perennial weeds were not assessed as they were very rare after two years of grass‐clover ley. In December 2014 and 2015 (assessment after potatoes only), all trays were placed in a heated greenhouse for two weeks to assess all seedlings before start of the frost period. This reduced the number of unknown species that had been high during the initial assessments in 2012 and 2013. In May, when the emergence rate was decreasing, the soil in the plastic trays was mixed to simulate field disturbance and to break compacted soil clods.

### 2.2 Data processing and statistical analysis

Emerged seedling densities from plastic trays were extrapolated to 1 m^2^ prior to analysis:

$${\text{Seeds m}}^{-2}=\text{seeds}\left(\text{tray}\right)\times \frac{10,000{\;\text{cm}}^{2}\times \text{volume}\left(\text{core}\right)}{\text{volume}(\text{tray})\times \text{area}(\text{core})}$$

Statistical analyses were performed with R version 3.4.2 (R Core Team, 2017), using the packages “nlme” (Pinheiro et al., 2017) and “vegan” (Oksanen et al., 2017) for multivariate analysis of data. Linear mixed effects models (lme) were performed on square‐root (*x* + 1)‐transformed number of seedlings per species in the seed bank (0–25 cm), total number of seedlings per soil layer, the comparison of the weed seed bank size at the start and the end of the experiment, and untransformed number of species (species richness). Fixed factors were tillage, crop rotation, and fertilizer while random factors were experiments, field replicates, tillage, and crop rotations, each nested in the preceding factor. Thus, the formula used in R was as follows:

lme(y∼tillage+crop rotation+fertilizer,random=∼1|experiment/replicate/tillage/crop rotation)

For the comparison of the weed seed bank density at the start and the end, the fixed factor was time (start, end of the experiment) while the random factors remained constant. Fixed factor interactions were included or eliminated depending on the model with the lowest Akaike information criterion (AIC) and Bayesian information criterion (BIC) fitted by the maximum log‐likelihood. The factors of the final model were further tested on variance homoscedasticity using Levene's test and fitted by maximizing the restricted log‐likelihood. The constant variance function “varIdent” was included in the model for each treatment with heteroscedastic distribution of residuals (Zuur, Leno, Walker, Saveliev, & Smith, 2009).

For multivariate data analyses, relative abundance indices (RAIs) were computed of the 15 major species or genera (>1% relative frequency in the seed bank at the end of the experiment) for each soil layer according to (Derksen, Lafond, Thomas, Loeppky, & Swanton, 1993) as (relative density + relative frequency)/2. The relative density is defined as the number of seedlings of a species relative to the total number of weed seedlings in percent. The relative frequency is obtained by dividing the absolute frequency of a species (number of samples where the species occurred/total number of samples) by the sum of absolute frequencies of all identified species in percent. The RAI corrects for patchy occurrences of weeds (Derksen, Lafond, Thomas, Loeppky, & Swanton, 1993) and, thus, generally improves the explanatory power of ordination techniques. Data were arcsine‐transformed to increase the variance homogeneity and improve the normal distribution prior to analysis.

**Figure 1:**
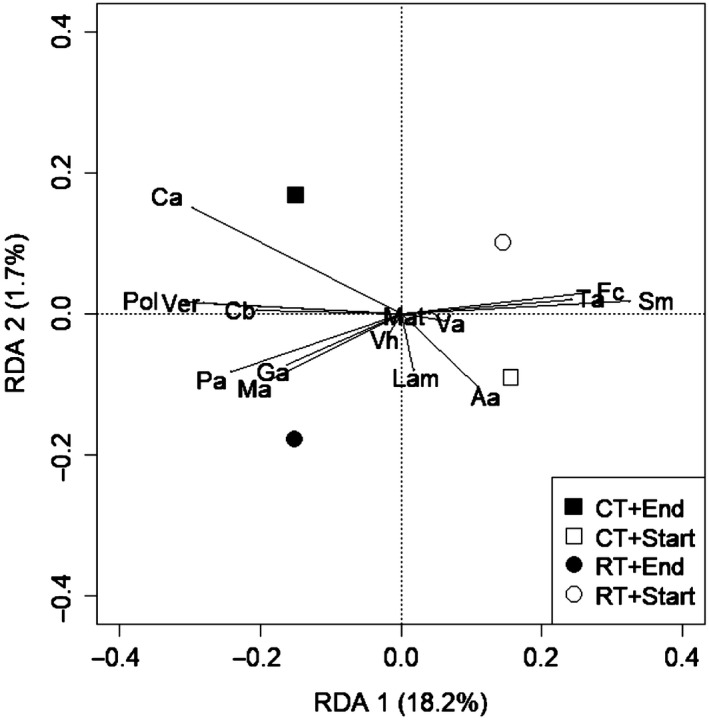
Redundancy analysis biplot comparing the weed seed bank community under noninversion (RT, circles) and conventional (CT, squares) tillage before wheat (start, unfilled symbols) and after the wheat–potato cropping sequence (end, filled symbols) in 0–12.5 cm depth. The first and second RDA axes are showing the proportion of explained eigenvalues by the factors after removing the variance caused by conditional variables (experiment, replicates). The 15 species (Aa = Aphanes* arvensis, *Cb = Capsella* bursa‐pastoris, *Ca = Chenopodium* album, *Fc = Fallopia* convolvulus*, Ga = Galium* aparine, *Lam = Lamium spp., Mat = Matricaria spp., Ma = Myosotis* arvensis*, Pa = Poa* annua*, Pol = Polygonum spp., Sm = Stellaria* media*, Ta = Thlaspi* arvense*, Vh = Veronica* hederifolia*, Ver = Veronica spp., and Va = Viola* arvensis*) with more than 1% frequency in the soil seed bank after potatoes are represented

The detrended correspondence analysis on RAI values revealed short gradients (<2) on the first axis suggesting the application of a redundancy analysis (RDA, (Dormann & Kühn, 2011)). The RDA was performed for each depth separately with all experimental factors and the factor “time” (start vs. end of the experiments, the covariates “experiment” (2012–2014, 2013–2015), the four replicates per experiment, and relative crop yields (start = wheat yields, end = potato yields) to account for soil heterogeneity. Covariates explained about 21% of the variance, which was removed from the total variance in the further analysis, thus increasing the overall power. Crop rotation and compost application had no statistically significant effect on the weed species composition dynamic according to permutation test (999 permutations) and were treated as covariates. The factors time, tillage, and their interaction were significant (*F*_1,247_ = 56.3, 2.7, and 5.2, respectively) according to permutation tests and, thus, combined in the RDA model (Figure 1).

## 3 RESULTS

The experimental factors rarely interacted with each other with respect to the species observed, and if, no general pattern was observed. For this reason, only main effects are shown (Table 2). In general, weed species that were frequent after the termination of grass‐clover, for example, *Aphanes arvensis, Chenopodium album, Matricaria* spp., and *Veronica* spp. (data not shown), also occurred at high densities after potatoes (Table 2).

**Table 2:** Seedling densities (sqrt(no. of seedlings m^−2^ + 1)) from seed bank tests of 15 major weed species and the total number of seedlings in 0–25 cm depth

		*Aphanes arvensis *L.	*Capsella bursa‐pastoris* (L.) Medicus	*Chenopodium album *L.	*Fallopia convolvulus *(L.) A. Loeve	*Galium aparine *L.	*Lamium spp.*	*Matricaria spp.*	*Myosotis arvensis *(L.) Hill.	*Poa annua *L.	*Polygonum spp.*	*Stellaria media *(L.) Vill.	*Thlaspi arvense *L.	*Veronica hederifolia *L.	*Veronica spp.*	*Viola arvensis *Murray	Total
sqrt(no. of seedlings m^−2 ^+ 1)																	
Tillage (T) (*df *= 7)	CT	20.7	20.2	34.1	11.9	5.8	15.4	23.8	15.1	9.7	12.6	11.5	14.7	16.7	40.4	4.8	93.7
	RT	23.8	22.5	29.0	14.6	11.9	22.5	17.0	19.9	13.9	12.8	9.8	14.6	17.6	45.6	4.9	98.5
	SED	3.17	2.21	3.15	2.47	1.81	2.68	5.30	1.67	3.77	2.87	1.81	2.36	3.40	4.19	1.64	4.70
	*p‐*Value					0.012	0.030		0.025								
Crop rotation (CR) (*df *= 15)	WF	22.2	22.8	30.7	14.2	8.3	18.0	20.7	16.9	13.3	13.0	10.7	14.4	18.4	43.4	5.4	96.8
	CC	22.2	19.9	32.3	12.3	9.4	19.9	20.1	18.1	10.4	12.4	10.7	14.9	15.8	42.6	4.2	95.4
	SED	2.07	1.71	2.06	1.69	1.74	1.58	2.12	2.02	2.70^b^	1.95	1.81	1.62	1.69	2.47	1.00	3.52^b^
	*p‐*Value																
Fertilizer (F) (*df *= 95)	+YWC	21.9	20.2	31.8	14.0	8.4	19.3	19.9	15.9	12.7	14.1	9.8	14.2	17.4	41.8	4.6	94.9
	−YWC	22.5	22.5	31.2	12.5	9.3	18.6	20.9	19.1	10.9	11.3	11.5	15.1	16.8	44.2	5.1	97.3
	SED	2.07	1.71	1.96	1.69	1.42	1.49	1.44	1.84^a^	1.91	1.95	1.53	1.62	2.61^a^	2.53	1.00	2.11
	*p‐*Value								0.001								

Note

Effects of conventional (CT) versus noninversion tillage (RT), two crop rotations consisting of wheat and potato with a weedy fallow (WF) versus cover crops sown after wheat (CC), and compost (+YWC) versus mineral phosphorous and potassium (−YWC) fertilization are shown. Exact *p*‐values (for *p* < 0.05) and standard errors of the difference between means (SED) result from linear mixed effects models (lme).

^a^ *df *= 94, ^b^ *df* = 14 as a result of CR × F and T × CR interactions included in the lme model, respectively.

Seedling densities (Table 2) and relative abundance indices (RAIs, data not shown) for the 15 major weed species were similar, although differences between factor levels were generally reduced for RAI. Overall, the 15 most common species that occurred at >1% relative frequency after potatoes were little affected by the applied treatments across the 0–25 cm soil layer (Table 2). Seedling densities of *Galium aparine *(*t*_7_ = 3.38, *p = *0.012), *Lamium* spp. (*t*_7_ = −2.71, *p = *0.03), and *M. arvensis* (*t*_7_ = 2.83, *p = *0.025) were significantly greater under RT than under CT. In contrast, *C. album* (*t*_7_ = −1.63, *p = *0.14) and *Matricaria* spp. (*t*_7_ = −1.34, *p = *0.22) seedling densities were somewhat lower under RT than CT.

Overall, subsidiary crops and compost did not substantially affect the weed species composition and the total number of viable seedlings in the seed bank (Table 2). Only *M. arvensis* was significantly less frequent in plots that had received compost than in plots without compost application (*t*_94_ = 3.46, *p < *0.001).

The weed species richness depended on soil depth and was only affected by tillage (Table 3). Hence, significantly more weed species were identified in the topsoil layer (0–12.5 cm, *t*_7_ = 2.92, *p = *0.02) under RT. In contrast, the number of weed species under RT was significantly reduced in the subsoil layer (12.5–25 cm, *t*_7_ = −2.54, *p = *0.04). No significant differences were observed when considering the 0–25 cm depth for both parameters (Table 3).

**Table 3:** Number of weed species (species richness) in each soil layer after the 4‐year crop rotation consisting of 2 years of grass‐clover, winter wheat, cover crops, and potato averaged over both field experiments

		Soil layer (cm)		
		0–12.5	12.5–25	0–25
No. of species				
Tillage (*df *= 7)	CT	8.4	7.8	11.3
	RT	9.9	6.7	12.1
	SED	0.52	0.43	0.38
	*p*‐Value	0.022	0.039	
Crop rotation (*df* = 15)	WF	9.2	7.3	11.9
	CC	9.0	7.1	11.5
	SED	0.42	0.31	0.32
	*p*‐Value			
Fertilizer (*df* = 95)	+YWC	8.9	7.3	11.7
	−YWC	9.3	7.1	11.7
	SED	0.31	0.30	0.28
	*p*‐Value			

Note

Applied treatments were noninversion (RT) versus conventional (CT) tillage after grass‐clover, weedy fallow (WF) versus cover crops (CC) sown after wheat, and compost (+YWC) versus mineral potassium and phosphorous application (−YWC). Exact *p*‐values (for *p* < 0.05) and standard errors of the difference between means (SED) result from linear mixed effects models (lme).

The redundancy analysis (RDA) showed a strong influence of the sampling time (start vs. end of the wheat–potato rotation, *F*_1,247_ = 56.26, *p < *0.001) and tillage (*F*_1,247_ = 2.67, *p = *0.012) on the weed seed bank community with a strong interaction effect of both factors (*F*_1,247_ = 5.17, *p < *0.001). Therefore, both treatments were merged for the 0–12.5 cm soil layer (Figure 1). At 12.5–25 cm depth, the effects of both factors followed the same pattern but were less clear (data not shown).

In the topsoil layer (0–12.5 cm, Figure 1), the first (RDA 1, *F*_1,247_ = 56.50, *p < *0.001) and second (RDA 2, *F*_1,247_ = 5.30, *p = *0.003) axis were significant at *p* < 0.01 with 18.2 and 1.7% explained eigenvalues, respectively. *Polygonum* spp., *C. album*, *Veronica* spp., and *P. annua* were associated with the end of the crop rotation, indicating an increase over time. In contrast, *S. media*, *F. convolvulus*, and *T. arvense* were plotted with the beginning of the crop rotation, indicating a decline over time. Most species were neither correlated with the positive nor negative side of the second RDA axis and, thus, unaffected by tillage. However, *C. album* grouped with CT at the end of the rotation, indicating that its increase over time was stronger under CT than RT. In contrast, *P. annua, M. arvensis*, and *G. aparine* grouped with RT at the end of the rotation, indicating that these species increased more under RT than CT over time. The strong interactions of tillage treatments with sampling time are illustrated by the closeness of RT and CT at the start of the crop rotation, while both parameters were clearly separated on the RDA 2 at the end of the crop rotation.

**Figure 2:**
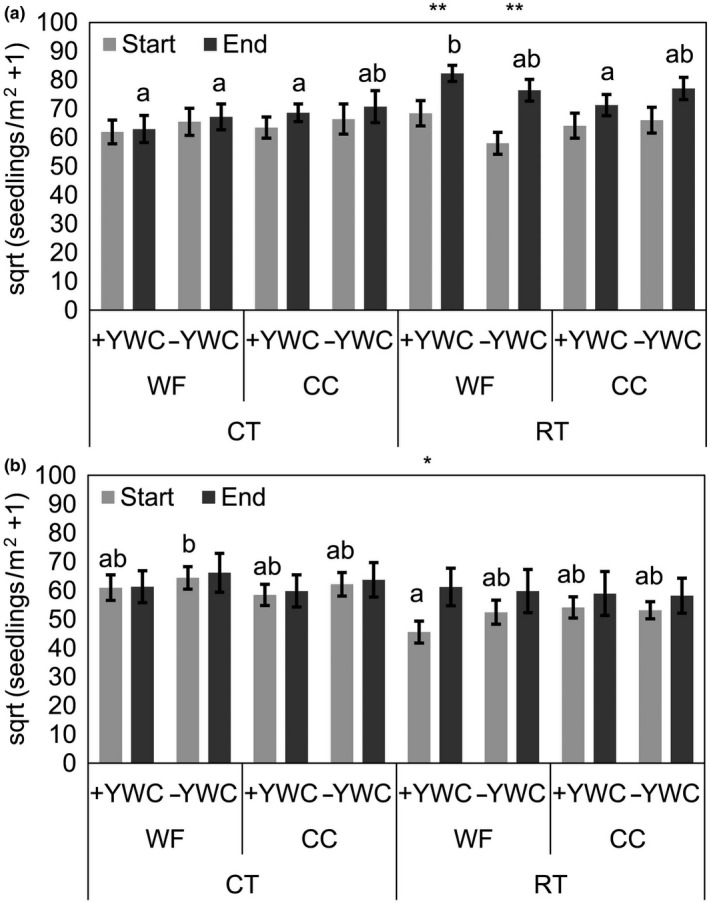
Total number (square‐root‐transformed) of weed seedlings m^−2^ (mean + *SE*) in the topsoil (a, 0–12.5 cm) and subsoil (b, 12.5–25 cm) layer before (light gray bars) and after (dark gray bars) the wheat–potato cropping sequence for each factor combination averaged over both experimental fields. Factor levels were conventional (CT) versus noninversion (RT) tillage, weedy fallow (WF) versus cover crops (CC), and compost application (+YWC) versus no compost (−YWC). Same lower‐case letters indicate not statistically different treatment combinations (lme followed by Tukey tests; *p* < 0.05; *df* = 89). *,** indicate statistically significant differences in seed bank size before (start) and after (end) the wheat–potato cropping sequence at *p* < 0.05 and *p* < 0.01 (lme, *df* = 23)

In the topsoil layer, no significant differences among any factor combinations were observed in the total weed seed bank before start of the wheat–potato cropping sequence (*F*_7,89_ = 1.15, *p* > 0.05, Figure 2a). In contrast, the factor combinations had a significant effect on the weed seed bank at the end of the cropping sequence (*F*_7,89_ = 3.41, *p = *0.003). The seed bank in the topsoil did not increase significantly over the course of the two years except for the RT treatment with the weedy fallow (*F*_1,23_ > 8.35, *p < *0.009, Figure 2a). In CT, at the end of the cropping sequence, weedy fallow and cover crops did not differ both in the presence and in the absence of YWC. In contrast, under RT, combining cover crops and yard waste compost led to significantly lower seedlings than after the weedy fallow with yard waste compost and, thus, kept the seed bank size at the same level than all CT treatments (Tukey‐HSD, *p < *0.05*, *Figure 2a).

In the subsoil layer, initial seed bank sizes after the first differential tillage had been applied were overall lower under RT than under CT, although most treatment combinations were not significantly different from each other (*F*_7,89_ = 1.70, *p = *0.12, Figure 2b). At the end of the cropping sequence, weed seed banks among all factor combinations were similar (*F*_7,89_ = 0.71, *p* > 0.05). Thus, apparently, weed seed banks increased under RT but not under CT with the strongest increase in the RT + weedy fallow + compost treatment (*F*_1,23_ = 6.49, *p = *0.018, Figure 2b).

## 4 DISCUSSION

Winter wheat and potatoes grown in rotation generally affect weed seed banks differently. Our results confirm an earlier study, where *G. aparine*, *Matricaria recutita, M. arvensis, P. annua, *and *Veronica *spp. increased in winter wheat and *C. album *and *Polygonum lapathifolia *increased in potato, while winter wheat and potatoes reduced *T. arvense *and *A. arvensis*, respectively (Albrecht, 2005). The large increase in frequency of *Veronica *spp. may be explained by the generally high persistence of the seeds and the variable seasonal emergence behavior of several *Veronica* species rendering the *Veronica *complex rather unaffected by rotations of spring and autumn sown crops (Clarke, Ginsburg, Kelly, & Tonguc, 2009).

### 4.1 Tillage determines weed seed bank size and community

The effect of RT on the weed seed bank can be variable (Nichols, Verhulst, Cox, & Govaerts, 2015), though increases in seed bank sizes and species richness compared to CT are commonly reported (Cardina, Herms, & Doohan, 2002; Carter & Ivany, 2006; Sosnoskie, Herms, & Cardina, 2006). Our results confirm the results of others (Bàrberi & Lo Cascio, 2001; Cardina et al., 2002; Nichols et al., 2015) that RT is concentrating weed seeds and species richness in the topsoil compared to CT while opposite effects occurred in the deeper soil layer. However, no overall increased seed bank at 0–25 cm was found. This is in contrast to the 14 years study of Carter and Ivany (2006). They observed greater weed seed banks in 0–10 and 10–20 cm after rotary harrowing (10 cm) and direct drilling than after moldboard ploughing (20 cm) and hypothesized that the fine loamy sand in their fields probably contributed to higher seed movement to deeper soil layers compared to clay‐textured soils. Thus, the overall increase in the seed bank at 12.5–25 cm under RT in our study is unexpected. It could be that potato harvesting homogenized the weed seeds down to at least 15 cm, partially camouflaging previous tillage effects on the weed seed bank.

Although the total weed seed bank under RT was higher than under CT, varying effects were observed at the species level. This is not unusual as germination requirements differ among weed species. For instance, less disturbed environments, such as present in RT and weedy fallow systems here, generally support the reproduction of *P. annua *(Davis, Renner, & Gross, 2005; Nichols et al., 2015) resulting in larger seed banks than in CT or cover cropping systems (Froud‐Williams, Chancellor, & Drennan, 1981; Wilson, Mascianica, Hines, & Walden, 1986). Furthermore, we observed significantly higher numbers of *G. aparine, Lamium *spp., and *M. arvensis* seedlings under RT compared with CT. This is in line with a French study, in which shallow ploughing resulted in fivefold and 25‐fold higher seed densities of *G. aparine *and *M. arvensis* in the top 10 cm soil*, *respectively, compared to deep ploughing (Dessaint, Chadoeuf, & Barralis, 1997).

The fact that *Matricaria *spp. seed banks were greater after CT than RT after potatoes may be best explained by the initially twofold higher seed density of *Matricaria *spp. in CT than in RT soils, which is also demonstrated by the low response of *Matricaria* spp. to tillage in the redundancy analysis.

In contrast, initial seedling densities of *C. album* were similar under CT than RT but in the redundancy analysis, a clear positive correlation was only shown between *C. album *and CT after the wheat–potato cropping sequence. The reproduction of *C. album *was likely directly affected by the dead mulch applied on RT plots directly after emergence of potatoes, resulting in almost 100% soil cover from mid‐May until mid‐July. In contrast, potatoes under CT were hilled until mid‐June, thus enabling seeds of *C. album *to germinate and reproduce afterward (Clarke et al., 2009). Similarly, 9 t/ha of a rye mulch provided excellent weed control in summer crops (Reberg‐Horton et al., 2012). Furthermore, *C. album *is particularly adapted to intensive tillage due to its strong dormancy and, thus, tends to be more prevalent under CT (Clements, Benott, Murphy, & Swanton, 1996).

### 4.2 Low effects of cover crops and compost on the weed seed bank

Subsidiary crops are grown predominantly for their ecosystem services, such as nitrogen uptake and supply, weed control, reduction of soil erosion and nitrogen leaching, and increase of soil fertility (Hartwig & Ammon, 2002; Radicetti et al., 2018). The establishment of living mulches (LM) in fall crops, such as winter wheat, can be critical in central Europe. Early sown winter hardy white clover species are generally very competitive and may reduce winter wheat yields (Carof, Tourdonnet, Saulas, Floch, & Roger‐Estrade, 2007). In contrast, late sowing may result in weak initial clover establishment leading to greater susceptibility to frost events and low competitiveness to the winter wheat. Probably for these reasons, LM in our study likely failed and were continued as weedy fallows after wheat in contrast to the cover crop plots. This further explains low differences for wheat‐associated weeds, for example, *G. aparine, Lamium *spp., *M. arvensis,* and *Veronica *spp., in the seed bank of weedy fallow and cover crop treatments as there was no suppression by LM. As discussed above, only *P. annua *and *C. bursa‐pastoris *seedlings tended to be higher in the weedy fallow than in cover crop plots. Both species flower throughout the year (Clarke et al., 2009) and could produce seeds in the weedy fallow until termination prior to planting potatoes. This is particularly important for *P. annua* which can live for at least two seasons in undisturbed soils and generally produces more seeds in the second year (Law, Bradshaw, & Putwain, 1977). In contrast, both species were disturbed after wheat harvest by cover crop sowing that was accompanied by shallow tillage.

Compost effects on weeds are generally more variable than those of LM depending on their initial composition (Blackshaw et al., 2005), composting duration, and methods (Cayuela, Millner, Meyer, & Roig, 2008). On the species level, we only found the wheat‐associated weed *M. arvensis *to be significantly reduced in compost‐amended plots while the potato‐associated weeds, such as *C. album *and *Polygonum *spp., increased. One can only speculate whether these differences were due to a different composting duration and technique as well as nutrient composition of the composts applied to the respective crops (Table 1). Other studies found high concentrations of volatile fatty acids and low C:N ratios in composts to be responsible for weed suppression (Blackshaw et al., 2005; Ozores‐Hampton et al., 2001).

### 4.3 RT with cover crops and compost did not buildup weed seed banks

The weed seed banks up to 15 cm depth generally correlate with the aboveground weed community (Dessaint et al., 1997; Rahman, James, & Grbavac, 2006) and can therefore be used to assess the efficiency of weed management systems. However, in the short term, specific crop management strategies, such as cover cropping, can lead to lower weed biomass than in systems without cover crops despite initially larger weed seed banks (Moonen & Bàrberi, 2004). In the long term, this may lead to lower weed seed banks in the cover crop system compared to the system without cover crops. The weed seedling density increased significantly in the weedy fallow under RT compared to CT or RT with cover crop treatments. The soil was shallowly tilled for cover crop sowing but not in the weedy fallow resulting from the failed LM, resulting in differences between these treatments. Thu, cover crop sowing was similar to a “stale seedbed” technique as weeds and volunteer wheat could germinate after harvest and were killed subsequently by tillage (Finckh & van Bruggen, 2015). Furthermore, long‐term organic management with frequent and deep soil tillage before start of the experiments may have resulted in weed seed banks whose seeds primarily require light for germination (Buhler, Hartzler, & Forcella, 1997). Direct drilling of cover crops after wheat via shallow undercutting could have minimized the weed seed exposure to light, probably maintaining weed seed dormancies. This implies that low weed seed banks can be maintained if management factors that increase (e.g., RT here) as well as decrease (e.g., cover crops and compost here) weed infestations are combined.

### 4.4 Future management implications

A recent meta‐analysis underlined that no‐tillage practices should be accompanied by crop rotation and residue retention to maintain crop yields compared with conventional tillage systems (Pittelkow et al., 2015). Our results indicate that in a herbicide‐free system, weed seed banks under RT with dead mulch application to potatoes could be maintained similar to weed seed banks under CT if cover crops and compost were used in addition in a wheat–potato cropping sequence. Thus, after two years of differential tillage, we found no evidence for increased annual weed pressure in well‐balanced RT systems. While simultaneous sowing of legume LM and winter wheat failed under our local conditions and, thus, failed to maintain weed seed banks as observed for cover crops, their potential for weed suppression should be further explored as in winter wheat alone the total weed seed bank of associated weeds increased considerably. For example, winter wheat sown in existing LM swards via strip tillage could be an alternative which deserves future attention. Important aspects here are earlier suppression of precrops as well as different widths of tillage strips at wheat sowing to balance intercrop as well as crop–weed competition.

## CONFLICT OF INTEREST

None declared.

## AUTHORS' CONTRIBUTION

S.J. and M.R.F. conceived the ideas and designed the experiment. S.J. and J.H.S. executed the experiment and collected the data, which was analyzed by J.H.S. J.H.S. and M.R.F. led the writing of the manuscript. All authors revised the drafts of the manuscript critically and approved the final manuscript for publication.

## Data Availability

Data supporting the results of the manuscript are available through Figshare: https://doi.org/10.6084/m9.figshare.7435565.
